# Clinical Characteristics and Surgical Outcomes of Bilateral Sequential Rhegmatogenous Retinal Detachment

**DOI:** 10.3390/jcm14228036

**Published:** 2025-11-13

**Authors:** Ida Gordon, Ndeye Coumba Ndiaye, Karine Angioi-Duprez, Jean-Paul Berrod, Jean-Baptiste Conart

**Affiliations:** 1 Department of Ophthalmology, CHRU-Nancy, Université de Lorraine, F-54000 Nancy, France; k.angioi-duprez@chru-nancy.fr (K.A.-D.); jp.berrod@chu-nancy.fr (J.-P.B.); j.conart@chru-nancy.fr (J.-B.C.); 2 UMR Inserm U1256 NGERE (Nutrition-Genetics and Exposure to Environmental Risks), Université de Lorraine, F-54000 Nancy, France; ndeye-coumba.ndiaye@univ-lorraine.fr; 3 Département Méthodologie, Promotion, Investigation, CHRU-Nancy, Université de Lorraine, F-54000 Nancy, France

**Keywords:** bilateral, rhegmatogenous retinal detachment, fellow eye, single surgery anatomical success, proliferative vitreoretinopathy

## Abstract

**Objectives:** To compare the clinical characteristics and surgical outcomes of initial and subsequent eyes in patients with sequential, bilateral rhegmatogenous retinal detachment (RRD). **Design:** Single-center observational retrospective cohort study. **Methods:** Sixty-eight patients who underwent surgery for sequential, bilateral RRD between January 2016 and December 2023 were included. Baseline characteristics, surgical procedures and postoperative outcomes were collected for both eyes. The primary outcome measure was the single-surgery anatomic success (SSAS), and the secondary outcome was final best-corrected visual acuity (BCVA). **Results:** Of the sixty-eight patients, 57 (83.8%) were male with a median age of 60.8 [55.1;69.0] years at first presentation. The median interval between RRD in the two eyes was 17.1 [11.5;33.5] months. Subsequent eyes presented with shorter symptom duration (*p* < 0.001), better baseline BCVA (*p* = 0.001), fewer quadrants involved (*p* < 0.001) and less frequent macular detachment (*p* = 0.004) compared with initial eyes. Preoperative grade B or C proliferative vitreoretinopathy (PVR) was observed in 33.8% of initial and 25.0% of subsequent eyes (*p* = 0.286). SSAS was achieved in 66.2% of initial and 73.5% of subsequent eyes (*p* = 0.458), with PVR as the main cause of failure (65.2% versus 61.1%, *p* = 1). The final median BCVA was similar in both eyes (0.1 [0.0;0.4] logMAR, *p* = 0.901). **Conclusions:** Although subsequent eyes were diagnosed earlier and presented with less advanced RRD, these advantages did not result in superior anatomical or functional outcomes. The high prevalence of PVR in both eyes likely accounts for these findings, supporting the hypothesis of a shared biological predisposition.

## 1. Introduction

Rhegmatogenous retinal detachment (RRD) remains a vision-threatening condition, despite significant advances in surgical techniques and anatomic success rates now approaching 90% [1,2]. In rare instances, RRD may involve both eyes, either simultaneously or sequentially, with potentially severe consequences for patients’ vision and quality of life, as well as considerable healthcare-related costs.

Established risk factors for RRD including myopia, pseudophakia and age-related vitreous changes typically affect both eyes [3]. Similarly, peripheral retinal degenerations and abnormalities of the vitreous base are often bilateral, a condition referred to as “fellow eye syndrome”, predisposing both eyes to retinal detachment [4,5]. Following unilateral RRD, the risk of fellow eye involvement is estimated to range from 3% to 13%, depending on study design and length of follow-up [3,4,5,6,7,8,9,10].

While the treatment and prognosis of unilateral RRD have been extensively documented in the literature, data on sequential bilateral RRD remain limited.

It can reasonably be assumed that patients with a history of RRD in one eye are more aware of the warning signs and are therefore more likely to present early if similar symptoms occur in the fellow eye. Several studies have thus shown that second eyes typically exhibit a shorter duration of symptoms, fewer macula-off detachments, and better baseline visual acuity [11,12,13,14]. However, surgical outcomes vary across studies and it remains unclear whether timely intervention results in improved anatomic and visual prognoses. Some authors have reported a higher reattachment rate in the subsequent eye while others have found no difference, suggesting that the result in the first eye may serve as a predictor for the second eye [11,12,13,14].

The aim of this study was to evaluate the clinical characteristics and surgical outcomes of the initial and subsequent eye in patients presenting with bilateral, sequential RRD.

## 2. Materials and Methods

### 2.1. Patients and Study Design

This single-center retrospective cohort study was conducted at the University Hospital of Nancy using medical records from January 2016 to December 2023. Sixty-eight patients who underwent surgery for bilateral sequential primary RRD were included. The study respected the tenets of the Declaration of Helsinki and was approved by the Ethics Committee of the Nancy hospital (approval no. 2024PI029, reference n°436). According to the Committee, this retrospective, non-interventional study did not require patient consent under the French “Loi Jardé.” Written informed consent for surgery was obtained from all patients prior to the procedure.

Inclusion criteria were as follows: (1) patients with bilateral sequential primary RRD treated within the 8-year study period; (2) a minimum follow-up period of 6 months after surgery. Exclusion criteria were as follows: (1) simultaneous bilateral RRD; (2) traumatic or tractional retinal detachment; (3) surgery performed outside the institution or the study period; (4) prior vitreoretinal surgery.

The first eye to develop RRD was defined as the initial eye, and the subsequently affected eye was designated as the subsequent eye.

A comprehensive ophthalmologic examination was obtained for all patients at baseline. It included an assessment of best-corrected visual acuity (BCVA) with projected-light Snellen charts, axial length measurement (AL) using IOLMaster (IOL Master; Carl Zeiss Meditec AG, Jena, Germany), and biomicroscopy with anterior segment and dilated fundus examination. An Amsler–Dubois scheme was systematically established for each patient, specifying the extent of the RD, the number of retinal breaks, the existence of vitreous hemorrhage, and preoperative proliferative vitreoretinopathy (PVR) grading according to Machemer et al. [15].

Peripheral retinal abnormalities in the subsequent eye, particularly lattice degeneration, were systematically investigated and treated with laser photocoagulation.

All surgical procedures were performed by two experienced surgeons (JPB and JBC) and consisted of either scleral buckling or three-port pars plana vitrectomy (PPV), combined or not with phacoemulsification and posterior chamber intraocular implantation. Internal tamponade with 25% SF_6_, 20% C_2_F_6_/air mixture, or silicone oil was used as appropriate.

The choice of surgical technique and tamponade was made according to the surgeon’s preference based on preoperative findings, in accordance with standard practice. Although surgical choices were left to each surgeon’s discretion because of the retrospective design, both surgeons followed comparable protocols, limiting inter-operator variability. Details of surgical technique and tamponade distribution by surgeon are summarized in Supplementary Table S1.

Postoperative follow-up visits were routinely scheduled within the first week after surgery, and then at 1 month and 6 months postoperatively. Additional examinations were provided when needed.

### 2.2. Pre-, Intra- and Postoperative Data

Pre- and intra-operative data included patient age and sex, previous prophylactic treatment in the fellow eye, duration of symptoms, AL, BCVA, lens status, RRD characteristics and type of surgery.

Post-operative data were primary and final anatomic success rate, causes of failure, subsequent cataract removal and BCVA.

### 2.3. Outcome Measures

The primary outcome of the study was the single-surgery anatomic success (SSAS), defined as retinal reattachment after a single operation. The secondary outcome was the BCVA at the last follow-up visit.

### 2.4. Statistical Analysis

Snellen visual acuity was converted to logarithm of the minimum angle of resolution (logMAR) units for analysis. Low-vision categories were converted using standard approximations (counting fingers = 2.0 logMAR, hand motion = 2.3 logMAR, light perception = 2.7 logMAR, and no light perception = 3.0 logMAR) [16]. Qualitative variables were described as frequency and percentage. Quantitative variables were reported as median and interquartile range [IQR]. Outcomes variables were compared across groups using the McNemar’s test or the Cochran’s Q test for qualitative data, and the Wilcoxon signed-rank test for quantitative data. The threshold for statistical significance was set at *p* < 0.05. Statistical analyses were performed using R version 4.4.0 (24 April 2024).

## 3. Results

From January 2016 to December 2023, 1486 patients underwent surgery for primary RRD. Among them, 102 presented with bilateral RRD, of whom 34 were excluded for the following reasons: RRD diagnosed prior to the inclusion period (n = 8), surgical repair at another institution (n = 4), history of vitreoretinal surgery (n = 2), traumatic retinal detachment in the fellow eye (n = 1), combined tractional and rhegmatogenous retinal detachment (n = 3), simultaneous bilateral RRD (n = 5), and follow-up period less than 6 months after the second eye surgery (n = 11). As a result, 68 patients were included in the study. The rate of bilateral sequential RRD was estimated to be 5.6% when considering patients operated elsewhere or with follow-up period less than 6 months.

### 3.1. Baseline Characteristics and Intraoperative Data

Baseline characteristics and intraoperative data are given in Table 1.

Out of 68 patients, 57 (83.8%) were male with a median age of 60.8 [55.1; 69.0] years at presentation. The median interval between RRD in either eye was 17.0 [11.8; 33.5] months.

The median duration of symptoms was significantly shorter in the subsequent eye (5.0 [3.0; 13.8] days versus 3.0 [2.0; 5.0] days, *p* < 0.001). Compared to the initial eye, the subsequent eye was more likely to have macula-on RRD (*p* = 0.004), fewer quadrants involved (*p* < 0.001), fewer breaks (*p* = 0.037) and better presenting BCVA (*p* = 0.001). Preoperative grade B or C PVR was observed in 23 (33.8%) of initial eyes and 17 (25.0%) of subsequent eyes (*p* = 0.286). There was no significant difference in lens status (*p* = 0.114) or in the presence of vitreous hemorrhage (*p* = 0.228).

The surgical approach involved PPV in almost all initial and subsequent eyes (*p* = 0.134), with no difference in the choice of internal tamponade (*p* = 0.819). Ten (14.7%) patients received prophylactic laser treatment of the fellow eye at the time of initial RRD.

### 3.2. Main Outcome Measure

Single-surgery anatomic success (SSAS) was comparable between the eyes, being achieved in 45 (66.2%) of initial eyes and in 50 (73.5%) of subsequent eyes (*p* = 0.458). SSAS rate among eyes that received prophylactic laser treatment prior to the initial retinal detachment repair was 70.0%.

The main cause of redetachment was the development of PVR, accounting for 15 out of 23 (65.2%) and 11 out of 18 (61.1%) failures in the initial and subsequent eyes, respectively (*p* = 1).

The final anatomic success was similar in both eyes (95.6%, *p* = 1).

### 3.3. Secondary Outcome

The median follow-up duration was 42.0 [23.5; 68.1] months.

The median BCVA significantly improved from 0.8 [0.2; 2.3] logMAR and 0.2 [0.0; 1.0] logMAR at baseline (*p* = 0.001) to 0.1 [0.0; 0.4] logMAR and 0.1 [0.0; 0.4] logMAR at the end of the follow-up in the initial and subsequent eyes, respectively. Final BCVA was comparable between the eyes (*p* = 0.901).

Sixteen (23.5%) initial and 14 (20.6%) subsequent eyes required cataract removal (*p* = 1). At the end of follow-up, there was no significant difference in lens status between the eyes (*p* = 0.683).

## 4. Discussion

This study provides additional insight into the clinical presentation and surgical outcomes of bilateral sequential RRD.

RRD is known to exhibit a marked bilateral tendency, with reported rates ranging from 3% to 13%, depending on study design, inclusion criteria, and follow-up duration [3,4,5,6,7,8,9,10]. The observed rate of 5.6% is in line with those reported by Walia et al. and Doukkali et al., who documented rates of 3.2% and 3.7% over 5 and 6 years, respectively [11,14].

Our cohort, predominantly composed of middle-aged myopic men, aligns with known epidemiological patterns [11,12,13,14]. The mean age of 61 years was slightly higher than in other studies (55–60 years), likely due to differences in patient characteristics, referral sources, or ocular comorbidities [11,12,13]. Simultaneous bilateral RRD, which tend to affect younger, more highly myopic patients, were excluded from our analysis to maintain homogeneity [14,17].

Consistent with previous studies, second-eye detachments in this cohort were diagnosed earlier, with less extensive involvement and better baseline visual acuity [11,12,13,14]. This likely reflects increased patient awareness following the initial RRD episode, as suggested by Govers et al. [18]. In their prospective study of patients treated for primary RRD, they showed that symptom awareness, often acquired through prior RRD experience, was associated with faster presentation, a higher rate of macula-on RRDs and improved postoperative visual outcomes [18]. Clinically, these results highlight the importance of promoting early detection strategies in at-risk individuals, as timely management remains the main determinant of macular preservation and visual prognosis.

Nevertheless, earlier detection and a more favorable initial presentation did not translate into better anatomic or functional outcomes in this cohort. The SSAS rate was slightly higher in subsequent eyes (73.5% versus 66.2%) but not statistically significant, and final BCVA was comparable. These findings are in agreement with those of Xu et al. and Doukkali et al., but differ from two other studies that reported improved outcomes in subsequent eyes, possibly due to earlier intervention and less advanced RRD at presentation [11,12,13,14].

The relatively lower SSAS rates observed in this series, compared to the 80–90% reported in other studies, may be attributed to differences in baseline characteristics, particularly the higher prevalence of PVR, which remains the leading cause of surgical failure [19]. PVR was present in 33.8% and 25.0% of initial and subsequent eyes, respectively, likely reflecting the tertiary referral nature of our center and delayed presentation in some cases. It accounted for more than half of all redetachments in both groups. This is consistent with the findings of Sheperd et al. who demonstrated that the development of postoperative PVR in one eye increased the risk in the fellow eye, supporting the notion of a bilateral predisposition [20]. The symmetry in PVR prevalence and recurrence patterns between eyes reinforces the concept of a ‘fellow eye syndrome’ and suggests a shared biological susceptibility that may outweigh the benefits of early surgical intervention.

Differences in anatomic outcomes across studies may also be influenced by variations in surgical technique. In the present series, the choice of procedure was guided by clinical factors such as lens status, the number, location, and size of retinal breaks, as well as the presence of PVR. Nearly all eyes underwent PPV, primarily due to pseudophakia or pre-existing PVR, without the addition of supplementary scleral buckle (SB), which may have affected the SSAS. In a paired-eye comparison, Xu et al. reported higher success rates with combined PPV and SB compared to PPV alone [12]. However, the benefit of this adjunctive technique in RRD repair remains debated [21,22,23]. In addition, there was no significant difference in the surgical approach between the initial and subsequent eyes, which reflects the underlying symmetry in baseline ocular characteristics and, importantly, minimizes potential bias in outcome comparison.

Finally, although evidence suggests that laser prophylaxis for lattice degeneration reduces the risk of fellow-eye RRD, its impact on surgical outcomes once detachment occurs remains uncertain [24,25,26]. In this cohort, ten (14.7%) patients received prophylactic laser treatment to the fellow eye at the time of initial RRD. Given the retrospective design, it was not possible to determine whether subsequent detachment was due to new retinal breaks or failure of previously treated areas. However, consistent with the findings of Xu et al., there was no significant difference in SSAS between eyes in this subgroup of patients, suggesting that prophylactic laser has limited influence on surgical prognosis [12]. Nevertheless, the small sample size precludes any definitive conclusions.

The present study has several limitations. First, its retrospective, single-center design inherently introduces selection and information biases, including potential inconsistencies in the documentation of clinical findings.

Second, some clinical details, such as the precise configuration of retinal breaks or the contribution of underlying connective tissue disorders, were not consistently available, precluding more detailed subgroup analyses. Third, although all surgeries were performed by experienced surgeons, the choice of surgical technique, tamponade, and use of adjunctive procedures were based on individual preference rather than a standardized protocol. In some cases, the two eyes were operated on by different surgeons, introducing potential inter-operator variability. Nevertheless, both surgeons followed comparable management principles, which likely minimized treatment-related bias. Finally, the specific characteristics of the bilateral sequential RRD population—particularly the high proportion of complex or PVR-associated cases referred to our center—may limit the generalizability of these findings to the broader RRD population.

Future research should aim to better identify modifiable factors influencing outcomes in bilateral sequential RRD. Larger and longer-term studies are needed to refine prognostic assessment and improve surgical success. Further exploration of patient-related and procedure-related contributors, as well as potential prophylactic measures and educational interventions, may help enhance visual outcomes in this population.

## 5. Conclusions

This study shows that the subsequent eye in bilateral sequential RRD is typically treated earlier and presents with less-advanced detachment. However, these initial advantages do not consistently translate into improved anatomic or functional outcomes. The high prevalence of PVR in both eyes likely contributes to the limited success rates and supports the hypothesis of a shared biological predisposition, consistent with the concept of fellow eye syndrome.

These findings highlight the importance of close surveillance of the fellow eye and timely management of new symptoms to preserve visual function. Further studies exploring preventive and educational strategies may help optimize outcomes in this high-risk population.

## Figures and Tables

**Table 1:** Baseline characteristics, pre- and intraoperative data of patients who were treated for bilateral sequential rhegmatogenous retinal detachment.

	First Eyes (n = 68)	Fellow Eyes (n= 68)	*p*-Value
Sex, n (%)			
Male	57 (83.8)		
Female	11 (16.2)		
Age, years (median [IQR])	60.8 [55.1; 69.0]	64.0 [57.0; 71.0]	0.001 ^a^
Axial length, mm (median [IQR])	25.4 [24.4; 26.5]	25.1 [24.5; 26.1]	0.891 ^a^
Preoperative lens status, n (%)			0.114 ^b^
Phakic	32 (47.1)	26 (38.2)	
Pseudophakic	36 (52.9)	42 (61.8)	
Preoperative BCVA, logMAR (median [IQR])	0.8 [0.2; 2.3]	0.2 [0.0; 1.0]	0.001 ^a^
Duration of RRD, days (median [IQR])	5.0 [3.0; 13.8]	3.0 [2.0; 5.0]	0.001 ^a^
Macular status, n (%)			0.004 ^b^
On	24 (35.3)	42 (61.8)	
Off	44 (64.7)	26 (38.2)	
Extent of RRD, number of quadrants (median [IQR])	2.0 [2.0; 3.0]	2.0 [1.8; 2.0]	<0.001 ^a^
Number of retinal tears (median [IQR])	2.0 [1.0; 3.0]	1.0 [1.0; 2.3)	0.037 ^a^
Preoperative PVR grade, n (%)			0.286 ^c^
None or grade A	45 (66.2)	51 (75.0)	
Grade B or C	23 (33.8)	17 (25.0)	
Vitreous hemorrhage, n (%)	11 (16.2)	6 (8.8)	0.228 ^b^
Surgical procedure, n (%)			0.134 ^b^
PPV	65 (95.6)	61 (89.7)	
SB	3 (4.4)	7 (10.3)	
Combined cataract extraction, n (%)	7 (10.3)	2 (2.9)	0.046 ^b^
Tamponade agent, n (%)			0.819 ^c^
SF6	51 (75.0)	53 (78.0)	
C2F6	8 (11.8)	6 (8.8)	
Silicon oil	6 (8.8)	2 (2.9)	
SB only (no tamponade)	3 (4.4)	7 (10.3)	

^a^ Wilcoxon signed-rank test, ^b^ McNemar test, ^c^ Cochran’s Q test. IQR = interquartile range, BCVA = Best-Corrected Visual Acuity, RRD = Rhegmatogenous Retinal Detachment, PVR = Proliferative Vitreoretinopathy.

## Data Availability

Data are available upon reasonable request from the corresponding author.

## Supplementary Materials

The following supporting information can be downloaded at: https://www.mdpi.com/article/10.3390/jcm14228036/s1, Table S1: Details of surgical technique and tamponade distribution by surgeon.

## Abbreviations

The following abbreviations are used in this manuscript:

AL	Axial Length
BCVA	Best-Corrected Visual Acuity
IQR	Interquartile Range
logMAR	Logarithm of the Minimum Angle of Resolution
PPV	Pars Plana Vitrectomy
PVR	Proliferative Vitreoretinopathy
RRD	Rhegmatogenous Retinal Detachment
SB	Scleral Buckling
SSAS	Single-Surgery Anatomic Success
