# Supplementary material for: Clinical Characteristics and Surgical Outcomes of Bilateral Sequential Rhegmatogenous Retinal Detachment
Source: J Clin Med. 2025 Nov 13;14(22):8036. doi: 10.3390/jcm14228036 (PMC12653507; doi:10.3390/jcm14228036)
Supplement: Supplementary file 1 [file jcm-14-08036-s001.zip › jcm-3937356-supplementary.pdf]

Table S1. Details of surgical technique and tamponade distribution by surgeon.

	Surgeon 1 (JPB)	Surgeon 2 (JBC)	p-value
**Number of eyes operated**, n (%)	69 (50.7)	67 (49.3)	
**Surgical technique**, n (%)			
- PPV	63 (91.3)	63 (94.0)	
- SB	6 (8.7)	4 (6.0)	
**Tamponade agent,** n (%)			
- SF6	52 (75.4)	52 (77.6)	
- C2F6	9 (13.0)	5 (7.5)	
- Silicon oil	2 (2.9)	6 (8.9)	
- SB only (no tamponade)	6 (8.7)	4 (6.0)	
**SSAS,** n (%)	47 (68.1)	48 (71.6)	

PPV = pars plana vitrectomy, SB = scleral buckling, SSAS = single-surgery anatomic success
